# The use of fulvestrant, a parenteral endocrine agent, in intestinal obstruction due to metastatic lobular breast carcinoma

**DOI:** 10.1186/1477-7819-6-128

**Published:** 2008-12-01

**Authors:** Jasmine YM Tang, Rajendra Singh Rampaul, Kwok L Cheung

**Affiliations:** 1 Division of Breast Surgery, University of Nottingham, Nottingham, UK

## Abstract

**Background:**

The role of fulvestrant in the management of intestinal obstruction associated with lobular carcinoma has not been specifically described.

**Case presentation:**

Herein we present two cases where fulvestrant, as the only available parenteral endocrine agent for postmenopausal advanced breast cancer has the opportunity to provide a means to initiate treatment in those patients who present with varying degrees of intestinal obstruction.

**Conclusion:**

Fulvestrant may obviate the use of chemotherapy while achieving sustained clinical benefit with less toxicity, in appropriately selected patients.

## Background

Fulvestrant (Faslodex) is a relatively new oestrogen receptor (ER) antagonist with a novel mode of action; it binds, blocks, and increases degradation of ER [1].

Fulvestrant is licensed for treatment of postmenopausal women with hormone receptor-positive advanced breast cancer (HR(+) ABC) progressing or recurring on anti-oestrogen therapy. However, it is also active in the first-line setting in patients with HR(+) tumours [1]. It is currently the only parenteral endocrine agent licensed for use in postmenopausal breast cancer, given as 250 mg intramuscularly every 4 weeks.

The role of fulvestrant in the management of intestinal obstruction associated with lobular carcinoma has not been specifically described. Herein we present two cases – both highlighting the use of fulvestrant in this context.

## Case presentation

### Case 1

An 82 year old lady presented as an emergency with small bowel obstruction but no history of abdominal surgery. Her chest X-ray revealed a small pleural effusion at the right base. Concomitantly, she was found to have a highly suspicious, palpable mass on her right breast.

**Figure 1:**
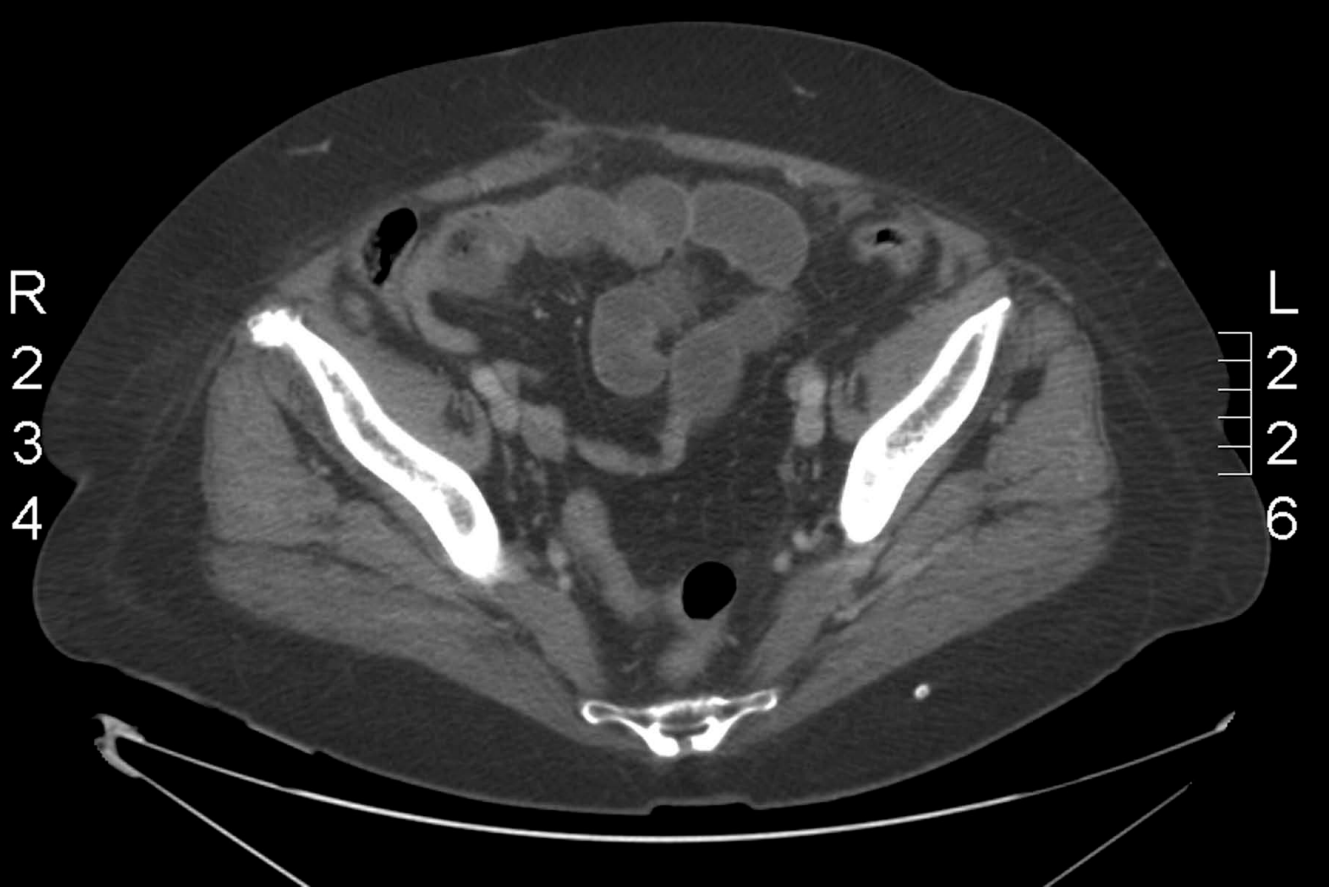
CT scan demonstrating mechanical small bowel obstruction.

CT scan findings revealed obstruction at the distal ileum (Figure 1), bilateral hydronephroses, widespread sclerotic bony metastases and a pulmonary embolus (PE). The right-sided breast mass was biopsied and this confirmed an invasive lobular adenocarcinoma (Grade 2), that was both strongly ER and progesterone receptor (PR) positive, with a H-score of 280 and 220 respectively.

She was deemed high risk for surgery due to her recent PE and she also did not wish to have surgery. In view of the circumstances, she was commenced on fulvestrant injections as a primary endocrine therapy.

This lady's intestinal obstruction eventually settled with non-operative management. When she was reviewed in the outpatient clinic two months after commencing fulvestrant, her tumour marker (CA15.3) had decreased from 57 to 38 kU/L. Follow-up CT scan at 6 months showed no evidence of progression of metastases with resolution of the small bowel obstruction.

At one year of fulvestrant, the overall assessment was that of a partial response with complete resolution of the palpable breast tumour.

### Case 2

With a background history of ER+ lobular breast carcinoma metastasizing to the lungs and bones for a few years, a 64 year old lady presented recently with symptoms of gastric outlet obstruction and changes in bowel habit.

This patient was first diagnosed with ER+ lobular breast carcinoma and was treated with wide local excision and post-operative radiotherapy. She then developed recurrences in her lymph node which progressed to her lungs and bones over the years.

**Figure 2:**
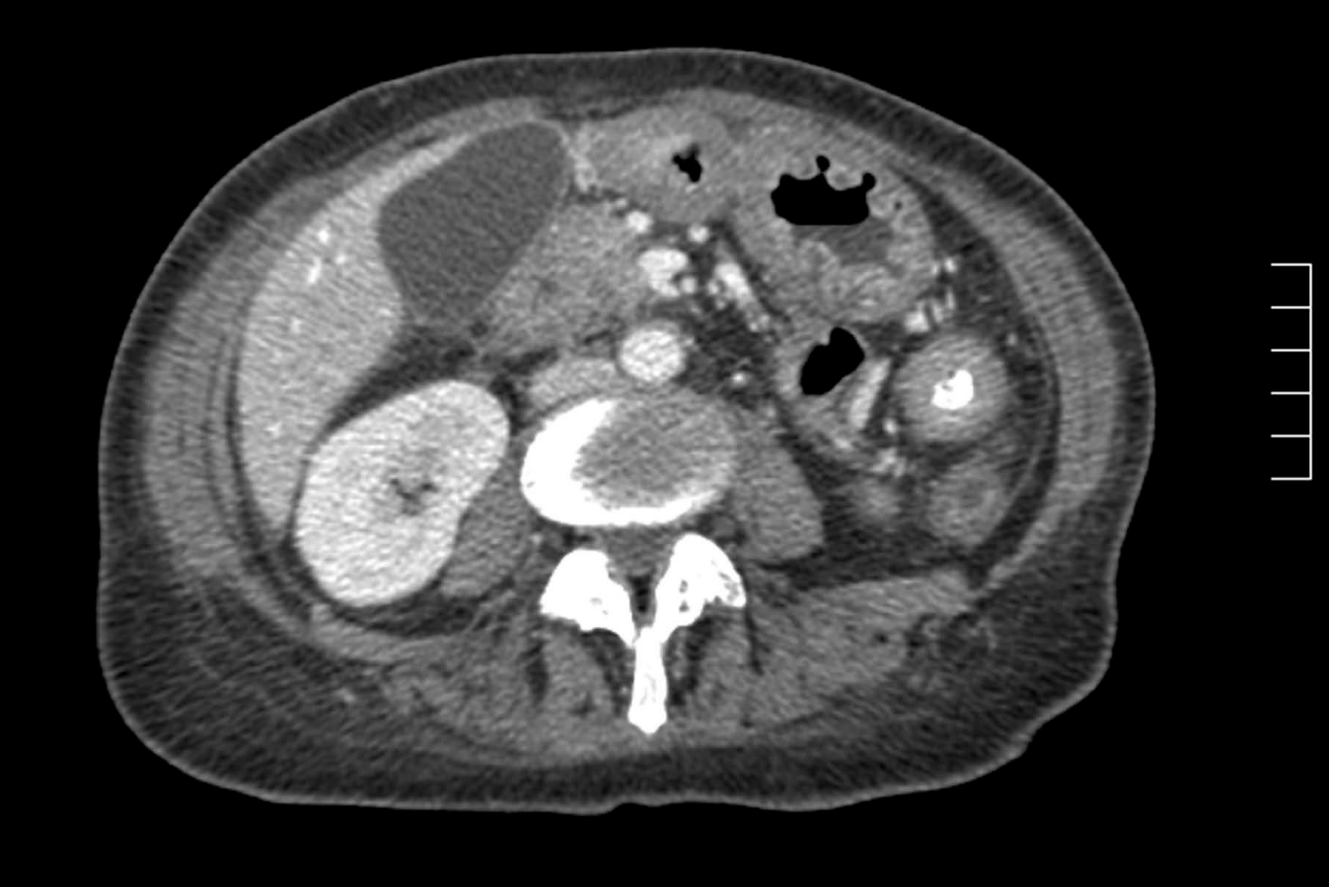
CT scan demonstrating thickening of colonic wall with narrowing of lumen.

CT scan revealed thickening in the duodenum and in both the ascending and descending colon with narrowing of the lumen (Figure 2). Biopsy results from both the duodenum and colon were consistent with metastases from a breast primary. Her symptoms of gastric outlet obstruction resolved after an uneventful gastrojejunostomy but her bowel symptoms remained. She was commenced on fulvestrant as systemic therapy following prior treatments with tamoxifen, then an aromatase inhibitor.

A repeat CT done 2 months later showed stable disease. She felt very well in herself with resolution of her bowel symptoms.

## Discussion

Lobular breast carcinoma accounts for about 8% to 14% of all breast cancers [2]. Several studies have demonstrated higher prevalence of spread of metastatic disease to the gastrointestinal tract, peritoneum and retroperitoneum, and ovaries in patients when compared to patients with ductal carcinoma [3,4]. Loss of expression of the cell-cell adhesion molecule E-cadherin in infiltrating lobular carcinoma may have contributed to these differences [5].

In hormone-responsive patients, endocrine therapy represents the mainstay of effective, well-tolerated treatment for advanced breast cancer before cytotoxic chemotherapy is required. A proviso for the success of any new endocrine therapy must be a lack of cross-resistance with prior treatments [6]. It is found that women who respond well to endocrine treatment for sustained periods tend to respond well to subsequent endocrine therapy. In Case 2, there was a decrease in the time lag between each endocrine therapy prior to starting fulvestrant. However, as noted, the patient responded well to treatment, obviating the need to commence chemotherapy.

This case report highlights not only the unusual presentation (ie intestinal obstruction) known to be associated with lobular carcinomas [2,5] but also the challenges this specific type poses to initiating therapy. In the presence of gastric metastasis, it is found that endocrine therapy (tamoxifen as a first line agent) is used as often as chemotherapy [7]. The chemotherapy schemes most frequently used were cyclophosphamide, methotrexate and 5 fluorouracil or cytoxan, doxorubicin and 5 fluorouracil. Initiating tamoxifen was not an option in Case 1 and fulvestrant proved to be an efficacious alternative.

A recent study demonstrated that fulvestrant was active in patients with multiple sites of metastases, visceral metastases, human epidermal growth factor receptor 2-positive disease and after heavy endocrine pre-treatment [8]. Another study comparing fulvestrant with anastrozole appears to show that patients with visceral metastases may have a longer duration of response with fulvestrant [9].

Two large randomized trials have previously shown that fulvestrant is at least as effective as anastrozole against breast cancer in postmenopausal women who failed on prior endocrine therapy [10,11]. However, fulvestrant showed neither superiority nor noninferiority in comparison to tamoxifen for the treatment of postmenopausal women who have received no prior hormonal or cytotoxic therapy for advanced breast cancer [12].

## Conclusion

Fulvestrant, is the only available parenteral endocrine agent for postmenopausal advanced breast cancer, and has the opportunity to provide a means to initiate treatment in patients who present with varying degrees of intestinal obstruction. This may obviate the use of chemotherapy while achieving sustained clinical benefit, with less toxicity, in appropriately selected patients.

## Consent

Written informed consent was obtained from the patient for publication of this case report and accompanying images. A copy of the written consent is available for review by the Editor-in-Chief of this journal.

## Competing interests

The authors declare that they have no competing interests.

## Authors' contributions

JYMT wrote the report, revised and submitted the manuscript for publication. KLC and RS helped with editing the report. All authors read and approved the final manuscript.
